# Supplementary material for: Brain activations associated with anticipation and delivery of monetary reward: A systematic review and meta-analysis of fMRI studies
Source: PLoS One. 2021 Aug 5;16(8):e0255292. doi: 10.1371/journal.pone.0255292 (PMC8341642; doi:10.1371/journal.pone.0255292)
Supplement: S1 File — (DOCX) [file pone.0255292.s002.docx]

**S1 File**

**1. Excluded studies**

*Note:* only includes studies using voxel-based analysis; data on other studies, eg ROI studies available from authors.

Breiter et al (2001)^1^

- Relevant data not reported; no response from authors

Knutson et al (2001a)^2^

- Scan did not cover whole brain

Knutson et al (2003)^3^

- Scan did not cover whole brain

Bjork et al (2004)^4^

- Scan did not cover whole brain

Elliott et al (2004)^5^

- Used block design that did not distinguish between anticipation and delivery

Knutson et al (2004)^6^

- Scan did not cover whole brain

Ramnani et al (2004)^7^

- Scan did not cover whole brain

Tanaka et al (2004)^8^

- Only report comparison between predictable and unpredictable reward conditions

Haruno et al (2004, 2006a,b)^9-11^

- No cue:reward contingency

Abler et al (2005)^12^

- Scan did not cover whole brain

Cox et al (2005)^13^

- Cue presented simultaneously with reward

Galvan et al (2005)^14^

- z-values not reported

Knutson et al (2005)^15^

- Modelled reward gain against reward loss

Small et al (2005)^16^

- No cue:reward contingency

Abler et al (2006)^17^

- Scan did not cover whole brain

Adcock et al (2006)^18^

- Scan did not cover whole brain

Daw et al (2006)^19^

- No data for anticipation; no response from authors about delivery data

Dreher et al (2006)^20^

- Paradigm employed in this study (differently from Dreher et al 2008) only measured expected frequency of reward ($20 in all cases).

Juckel et al (2006a,b)^21,22^

- Scan did not cover whole brain

Kirsch et al (2006)^23^

- Compared monetary reward to verbal feedback
- Only provide combined results for placebo and drug conditions

Yacubian et al (2006)^24^

- Scan did not cover whole brain

Abler et al (2007)^25^

- Scan did not cover whole brain

Kirsch et al (2007)^26^

- Does not report data for healthy controls

Schlagenhauf et al (2007)^27^

- Scan did not cover whole brain

Schott et al (2007)^28^

- Relevant data only reported in figure, no response from authors

Wrase et al (2007a,b)^29,30^

- Scan did not cover whole brain

Abler et al (2008)^31^

- Scan did not cover whole brain

Cooper et al (2008)^32^

- Modeled reward gain against reward loss

Dillon et al (2008)^33^

- z-values not provided

Elliott et al (2008)^34^

- Complicated task; not clear if models reward anticipation
- Only results for ROI analysis reported

Rolls et al (2008)^35^

- No cue:reward contingency

Schott et al (2008)^36^

- Relevant data not reported in paper; no response from authors

Strohle et al (2008)^37^

- Scan did not cover whole brain

Pleger et al (2008, 2009)^38,39^

- No cue:reward contingency

Abler et al (2009)^40^

- Scan did not cover whole brain

Beck et al (2009)^41^

- Scan did not cover whole brain

Carter et al (2009)^42^

- Scan did not cover whole brain

Engelmann et al (2009)^43^

- Complex design that did not separate reward anticipation from reward delivery

Plichta et al (2009)^44^

- No cue:reward contingency

Schlagenhauf et al (2009)^45^

- Scan did not cover whole brain

Spreckelmeyer et al (2009)^46^

- Scan did not cover whole brain

Walter et al (2009)^47^

- Scan did not cover whole brain

Buckholtz et al (2010)^48^

- Scan did not cover whole brain

Daniel et al (2010)^49^

- Compared monetary reward to cognitive feedback

Eisenberger et al (2010)^50^

- Participants did not receive winnings
- Findings on placebo not reported separately

Forbes et al (2010)^51^

- Sample consisted of adolescents

Koch et al (2010)^52^

- No cue:reward contingency

Hermans et al (2010)^53^

- Does not report data for placebo group

Koch et al (2010)^52^

- Study potentially includeable, but there were no activations during relevant task

Rademacher et al (2010)^54^

- Scan did not cover whole brain

Samanez-Larkin et al (2010)^55^

- Modelled reward gain against reward loss

Schonberg et al (2010)^56^

- Participants did not receive money in exchange for winnings

Waltz et al (2010)^57^

- Modelled reward gain against reward loss
- Does not report data for controls

Guitart-Masip et al (2010, 2011, 2012)^58-60^

- Scan did not cover whole brain

Andrews et al (2011)^61^

- Only reports differences between participants with and without a family history of alcoholism

Diaconescu et al (2011)^62^

- Does not report data for healthy controls

Figee et al (2011)^63^

- Scan did not cover whole brain

Jia et al (2011)^64^

- Does not report data for healthy controls

Jocham et al (2011)^65^

- Does not report anticipation data, no response from authors for delivery data

Kim et al (2011)^66^

- Modelled reward gain vs reward loss

Krebs et al (2011)^67^

- Scan did not cover whole brain

Mowrer et al (2011)^68^

- Did not separate reward from loss avoidance in analysis

Smoski et al (2011)^69^

- Relevant data not available in paper, authors unable to locate

Samanez-Larkin et al (2011)^70^

- Sample overlaps with Samanez-Larkin et al (2010)^55^ (above), but no further usable data

Da Silva Alves (2011, 2013), Van Duin et al (2016)^71-73^

- Participants did not receive money in exchange for winnings

Choi et al (2012)^74^

- Data for healthy controls not reported separately

Delmonte et al (2012)^75^

- Only provides data for monetary and social reward combined

Dichter et al (2012)^76^

- Does not report data for healthy controls

Dowd et al (2012)^77^

- Does not report data for healthy controls

Enzi et al (2012)^78^

- Scan did not cover whole brain

Esslinger et al (2012)^79^

- Only compare monetary reward to verbal reward

Juckel et al (2012)^80^

- Scan did not cover whole brain

Morris et al (2012)^81^

- Participants did not receive money in exchange for winnings

Nielsen et al (2012)^82^

- Modelled reward gain vs reward loss

Plichta et al (2012)^83^

- Compared monetary reward to verbal feedback

Puschmann et al (2012)^84^

- Only report non-usable time x learning interaction

Stoy et al (2012)^85^

- Scan did not cover whole brain

Weis et al (2012)^86^

- Scan did not cover whole brain

Balodis et al (2013)^87^

- Does not report data for healthy controls

Edel et al (2013)^88^

- Scan did not cover whole brain

Enzi et al (2013)^89^

- Relevant data only provided in figure, no response from authors

Kappel et al (2013)^90^

- Participants did not receive money for winnings (only a bonus for reaching a predetermined number of points)

Patel et al (2013)^91^

- Does not report data for healthy controls

Plichta et al (2013)^92^

- Compared monetary reward to verbal feedback

Roiser et al (2013)^93^

- Does not report data for healthy controls

Treadway et al (2013)^94^

- Scan did not cover whole brain

Balodis et al (2013, 2014)^87,95^

- Does not report data for healthy controls

Boecker et al (2014)^96^

- Only compares monetary to verbal reward

Grimm et al (2014)^97^

- Compared monetary reward anticipation to verbal feedback

Pfabigan et al (2014)^98^

- Scan did not cover whole brain

Rademacher et al (2014)^99^

- Participants didn’t receive money in exchange for winnings
- Scan did not cover whole brain

Spati et al (2014)^100^

- No cue:reward contingency

De Leeuw et al (2015)^101^

- Relevant data only reported in a figure, no response from authors

Enzi et al (2015)^102^

- Does not report data for healthy controls

Hagele et al (2015)^103^

- Does not report data for healthy controls

Hanssen et al (2015)^104^

- Does not report data for healthy controls

Metereau et al (2015)^105^

- Voxel-based data only reported for unsigned expected value

Redlich et al (2015)^106^

- Does not report data for healthy controls

Spaniol et al (2015)^107^

- Compared reward with loss anticipation
- Only reports combined data for elderly and adult groups

Subramaniam et al (2015)^108^

- Data for healthy controls only reported in a figure. Authors contacted; no reply

Balodis et al (2016)^109^

- Does not report data for healthy controls

Kirschner et al (2016)^110^

- Does not report measure of reward outcome (only ‘adaptive coding’)

Mori et al (2016)^111^

- Does not report data for healthy controls

Vink et al (2016)^112^

- Sample made up of adolescents

Garrison et al (2017)^113^

- Sample mainly adolescents

Richey et al (2017)^114^

- Modelled reward gain against reward loss
- Does not separate monetary and social reward

Verdejo-Roman et al (2017)^115^

- Not stated if participants received payment, no response from authors

Kocsel et al (2017)^116^ Kocsel et al (2019)^117^

- Participants did not receive money in exchange for winnings (participated in ‘prize draw’)

Dubol et al (2018)^118^

- Sample made up of a mixture of healthy subjects plus those with schizophrenia, depression and cocaine abuse

Held-Poschardt et al (2018)^119^

- Scan did not cover whole brain

Li et al (2018)^120^

- Does not report data for healthy controls

Spetter et al (2018)^121^

- Only reports findings for reward and loss anticipation combined

Yousuf et al (2018)^122^

- No cue:reward contingency
- Food and monetary reward not separated in the analysis

Just et al (2019)^123^

- Does not report data for healthy controls

Mori et al (2019)^124^

- Pooled data from three datasets with different scanning parameters

**References**

1. Breiter HC, Aharon I, Kahneman D, Dale A, Shizgal P. Functional imaging of neural responses to expectancy and experience of monetary gains and losses. *Neuron* 2001; **30**: 619-39.

2. Knutson B, Adams CM, Fong GW, Hommer D. Anticipation of increasing monetary reward selectively recruits nucleus accumbens. *Journal of Neuroscience* 2001; **21**: RC159.

3. Knutson B, Fong GW, Bennett SM, Adams CM, Hommer D. A region of mesial prefrontal cortex tracks monetarily rewarding outcomes: characterization with rapid event-related fMRI. *NeuroImage* 2003; **18**: 263-72.

4. Bjork JM, Knutson B, Fong GW, Caggiano DM, Bennett SM, Hommer DW. Incentive-elicited brain activation in adolescents: similarities and differences from young adults. *Journal of Neuroscience* 2004; **24**: 1793-802.

5. Elliott R, Newman JL, Longe OA, William Deakin JF. Instrumental responding for rewards is associated with enhanced neuronal response in subcortical reward systems. *NeuroImage* 2004; **21**: 984-90.

6. Knutson B, Bjork JM, Fong GW, Hommer D, Mattay VS, Weinberger DR. Amphetamine modulates human incentive processing. *Neuron* 2004; **43**: 261-9.

7. Ramnani N, Elliott R, Athwal BS, Passingham RE. Prediction error for free monetary reward in the human prefrontal cortex. *NeuroImage* 2004; **23**: 777-86.

8. Tanaka SC, Doya K, Okada G, Ueda K, Okamoto Y, Yamawaki S. Prediction of immediate and future rewards differentially recruits cortico-basal ganglia loops. *Nature Neuroscience* 2004; **7**: 887-93.

9. Haruno M, Kuroda T, Doya K, et al. A neural correlate of reward-based behavioral learning in caudate nucleus: a functional magnetic resonance imaging study of a stochastic decision task. *Journal of Neuroscience* 2004; **24**: 1660-5.

10. Haruno M, Kawato M. Different neural correlates of reward expectation and reward expectation error in the putamen and caudate nucleus during stimulus-action-reward association learning. *Journal of Neurophysiology* 2006; **95**: 948-59.

11. Haruno M, Kawato M. Heterarchical reinforcement-learning model for integration of multiple cortico-striatal loops: fMRI examination in stimulus-action-reward association learning. *Neural Networks* 2006; **19**: 1242-54.

12. Abler B, Walter H, Erk S. Neural correlates of frustration. *Neuroreport* 2005; **16**: 669-72.

13. Cox SM, Andrade A, Johnsrude IS. Learning to like: a role for human orbitofrontal cortex in conditioned reward. *Journal of Neuroscience* 2005; **25**: 2733-40.

14. Galvan A, Hare TA, Davidson M, Spicer J, Glover G, Casey BJ. The role of ventral frontostriatal circuitry in reward-based learning in humans. *Journal of Neuroscience* 2005; **25**: 8650-6.

15. Knutson B, Taylor J, Kaufman M, Peterson R, Glover G. Distributed neural representation of expected value. *Journal of Neuroscience* 2005; **25**: 4806-12.

16. Small DM, Gitelman D, Simmons K, Bloise SM, Parrish T, Mesulam MM. Monetary incentives enhance processing in brain regions mediating top-down control of attention. *Cerebral Cortex* 2005; **15**: 1855-65.

17. Abler B, Walter H, Erk S, Kammerer H, Spitzer M. Prediction error as a linear function of reward probability is coded in human nucleus accumbens. *NeuroImage* 2006; **31**: 790-5.

18. Adcock RA, Thangavel A, Whitfield-Gabrieli S, Knutson B, Gabrieli JD. Reward-motivated learning: mesolimbic activation precedes memory formation. *Neuron* 2006; **50**: 507-17.

19. Daw ND, O'Doherty JP, Dayan P, Seymour B, Dolan RJ. Cortical substrates for exploratory decisions in humans. *Nature* 2006; **441**: 876-9.

20. Dreher JC, Kohn P, Berman KF. Neural coding of distinct statistical properties of reward information in humans. *Cerebral Cortex* 2006; **16**: 561-73.

21. Juckel G, Schlagenhauf F, Koslowski M, et al. Dysfunction of ventral striatal reward prediction in schizophrenic patients treated with typical, not atypical, neuroleptics. *Psychopharmacology* 2006; **187**: 222-8.

22. Juckel G, Schlagenhauf F, Koslowski M, et al. Dysfunction of ventral striatal reward prediction in schizophrenia. *NeuroImage* 2006; **29**: 409-16.

23. Kirsch P, Reuter M, Mier D, et al. Imaging gene-substance interactions: the effect of the DRD2 TaqIA polymorphism and the dopamine agonist bromocriptine on the brain activation during the anticipation of reward. *Neuroscience Letters* 2006; **405**: 196-201.

24. Yacubian J, Glascher J, Schroeder K, Sommer T, Braus DF, Buchel C. Dissociable systems for gain- and loss-related value predictions and errors of prediction in the human brain. *Journal of Neuroscience* 2006; **26**: 9530-7.

25. Abler B, Erk S, Walter H. Human reward system activation is modulated by a single dose of olanzapine in healthy subjects in an event-related, double-blind, placebo-controlled fMRI study. *Psychopharmacology* 2007; **191**: 823-33.

26. Kirsch P, Ronshausen S, Mier D, Gallhofer B. The influence of antipsychotic treatment on brain reward system reactivity in schizophrenia patients. *Pharmacopsychiatry* 2007; **40**: 196-8.

27. Schlagenhauf F, Juckel G, Koslowski M, et al. Reward system activation in schizophrenic patients switched from typical neuroleptics to olanzapine. *Psychopharmacology* 2008; **196**: 673-84.

28. Schott BH, Niehaus L, Wittmann BC, et al. Ageing and early-stage Parkinson's disease affect separable neural mechanisms of mesolimbic reward processing. *Brain* 2007; **130**: 2412-24.

29. Wrase J, Kahnt T, Schlagenhauf F, et al. Different neural systems adjust motor behavior in response to reward and punishment. *NeuroImage* 2007; **36**: 1253-62.

30. Wrase J, Schlagenhauf F, Kienast T, et al. Dysfunction of reward processing correlates with alcohol craving in detoxified alcoholics. *NeuroImage* 2007; **35**: 787-94.

31. Abler B, Greenhouse I, Ongur D, Walter H, Heckers S. Abnormal reward system activation in mania. *Neuropsychopharmacology* 2008; **33**: 2217-27.

32. Cooper JC, Knutson B. Valence and salience contribute to nucleus accumbens activation. *NeuroImage* 2008; **39**: 538-47.

33. Dillon DG, Holmes AJ, Jahn AL, Bogdan R, Wald LL, Pizzagalli DA. Dissociation of neural regions associated with anticipatory versus consummatory phases of incentive processing. *Psychophysiology* 2008; **45**: 36-49.

34. Elliott R, Agnew Z, Deakin JF. Medial orbitofrontal cortex codes relative rather than absolute value of financial rewards in humans. *European Journal of Neuroscience* 2008; **27**: 2213-8.

35. Rolls ET, McCabe C, Redoute J. Expected value, reward outcome, and temporal difference error representations in a probabilistic decision task. *Cerebral Cortex* 2008; **18**: 652-63.

36. Schott BH, Minuzzi L, Krebs RM, et al. Mesolimbic functional magnetic resonance imaging activations during reward anticipation correlate with reward-related ventral striatal dopamine release. *Journal of Neuroscience* 2008; **28**: 14311-9.

37. Strohle A, Stoy M, Wrase J, et al. Reward anticipation and outcomes in adult males with attention-deficit/hyperactivity disorder. *NeuroImage* 2008; **39**: 966-72.

38. Pleger B, Blankenburg F, Ruff CC, Driver J, Dolan RJ. Reward facilitates tactile judgments and modulates hemodynamic responses in human primary somatosensory cortex. *Journal of Neuroscience* 2008; **28**: 8161-8.

39. Pleger B, Ruff CC, Blankenburg F, Kloppel S, Driver J, Dolan RJ. Influence of dopaminergically mediated reward on somatosensory decision-making. *PLoS Biology* 2009; **7**: e1000164.

40. Abler B, Herrnberger B, Gron G, Spitzer M. From uncertainty to reward: BOLD characteristics differentiate signaling pathways. *BMC Neuroscience* 2009; **10**: 154.

41. Beck A, Schlagenhauf F, Wustenberg T, et al. Ventral striatal activation during reward anticipation correlates with impulsivity in alcoholics. *Biological Psychiatry* 2009; **66**: 734-42.

42. Carter RM, Macinnes JJ, Huettel SA, Adcock RA. Activation in the VTA and nucleus accumbens increases in anticipation of both gains and losses. *Frontiers in Behavioral Neuroscience* 2009; **3**: 21.

43. Engelmann JB, Damaraju E, Padmala S, Pessoa L. Combined effects of attention and motivation on visual task performance: transient and sustained motivational effects. *Frontiers in Human neuroscience* 2009; **3**: 4.

44. Plichta MM, Vasic N, Wolf RC, et al. Neural hyporesponsiveness and hyperresponsiveness during immediate and delayed reward processing in adult attention-deficit/hyperactivity disorder. *Biological Psychiatry* 2009; **65**: 7-14.

45. Schlagenhauf F, Sterzer P, Schmack K, et al. Reward feedback alterations in unmedicated schizophrenia patients: relevance for delusions. *Biological Psychiatry* 2009; **65**: 1032-9.

46. Spreckelmeyer KN, Krach S, Kohls G, et al. Anticipation of monetary and social reward differently activates mesolimbic brain structures in men and women. *Social Cognitive and Affective Neuroscience* 2009; **4**: 158-65.

47. Walter H, Kammerer H, Frasch K, Spitzer M, Abler B. Altered reward functions in patients on atypical antipsychotic medication in line with the revised dopamine hypothesis of schizophrenia. *Psychopharmacology* 2009; **206**: 121-32.

48. Buckholtz JW, Treadway MT, Cowan RL, et al. Mesolimbic dopamine reward system hypersensitivity in individuals with psychopathic traits. *Nature Neuroscience* 2010; **13**: 419-21.

49. Daniel R, Pollmann S. Comparing the neural basis of monetary reward and cognitive feedback during information-integration category learning. *Journal of Neuroscience* 2010; **30**: 47-55.

50. Eisenberger NI, Berkman ET, Inagaki TK, Rameson LT, Mashal NM, Irwin MR. Inflammation-induced anhedonia: endotoxin reduces ventral striatum responses to reward. *Biological Psychiatry* 2010; **68**: 748-54.

51. Forbes EE, Olino TM, Ryan ND, et al. Reward-related brain function as a predictor of treatment response in adolescents with major depressive disorder. *Cognitivem Affective and Behavioral Neuroscience* 2010; **10**: 107-18.

52. Koch K, Schachtzabel C, Wagner G, et al. Altered activation in association with reward-related trial-and-error learning in patients with schizophrenia. *NeuroImage* 2010; **50**: 223-32.

53. Hermans EJ, Bos PA, Ossewaarde L, Ramsey NF, Fernandez G, van Honk J. Effects of exogenous testosterone on the ventral striatal BOLD response during reward anticipation in healthy women. *NeuroImage* 2010; **52**: 277-83.

54. Rademacher L, Krach S, Kohls G, Irmak A, Grunder G, Spreckelmeyer KN. Dissociation of neural networks for anticipation and consumption of monetary and social rewards. *NeuroImage* 2010; **49**: 3276-85.

55. Samanez-Larkin GR, Kuhnen CM, Yoo DJ, Knutson B. Variability in nucleus accumbens activity mediates age-related suboptimal financial risk taking. *Journal of Neuroscience* 2010; **30**: 1426-34.

56. Schonberg T, O'Doherty JP, Joel D, Inzelberg R, Segev Y, Daw ND. Selective impairment of prediction error signaling in human dorsolateral but not ventral striatum in Parkinson's disease patients: evidence from a model-based fMRI study. *NeuroImage* 2010; **49**: 772-81.

57. Waltz JA, Schweitzer JB, Ross TJ, et al. Abnormal responses to monetary outcomes in cortex, but not in the basal ganglia, in schizophrenia. *Neuropsychopharmacology* 2010; **35**: 2427-39.

58. Guitart-Masip M, Fuentemilla L, Bach DR, et al. Action dominates valence in anticipatory representations in the human striatum and dopaminergic midbrain. *Journal of Neuroscience* 2011; **31**: 7867-75.

59. Guitart-Masip M, Bunzeck N, Stephan KE, Dolan RJ, Duzel E. Contextual novelty changes reward representations in the striatum. *Journal of Neuroscience* 2010; **30**: 1721-6.

60. Guitart-Masip M, Chowdhury R, Sharot T, Dayan P, Duzel E, Dolan RJ. Action controls dopaminergic enhancement of reward representations. *Proceedings of the National Academy of Sciences of the United States of America* 2012; **109**: 7511-6.

61. Andrews MM, Meda SA, Thomas AD, et al. Individuals family history positive for alcoholism show functional magnetic resonance imaging differences in reward sensitivity that are related to impulsivity factors. *Biological Psychiatry* 2011; **69**: 675-83.

62. Diaconescu AO, Jensen J, Wang H, et al. Aberrant Effective Connectivity in Schizophrenia Patients during Appetitive Conditioning. *Frontiers in Human Neuroscience* 2011; **4**: 239.

63. Figee M, Vink M, de Geus F, et al. Dysfunctional reward circuitry in obsessive-compulsive disorder. *Biological Psychiatry* 2011; **69**: 867-74.

64. Jia Z, Worhunsky PD, Carroll KM, et al. An initial study of neural responses to monetary incentives as related to treatment outcome in cocaine dependence. *Biological Psychiatry* 2011; **70**: 553-60.

65. Jocham G, Klein TA, Ullsperger M. Dopamine-mediated reinforcement learning signals in the striatum and ventromedial prefrontal cortex underlie value-based choices. *Journal of Neuroscience* 2011; **31**: 1606-13.

66. Kim H, Shimojo S, O'Doherty JP. Overlapping responses for the expectation of juice and money rewards in human ventromedial prefrontal cortex. *Cerebral Cortex* 2011; **21**: 769-76.

67. Krebs RM, Boehler CN, Roberts KC, Song AW, Woldorff MG. The involvement of the dopaminergic midbrain and cortico-striatal-thalamic circuits in the integration of reward prospect and attentional task demands. *Cerebral Cortex* 2012; **22**: 607-15.

68. Mowrer SM, Jahn AA, Abduljalil A, Cunningham WA. The value of success: acquiring gains, avoiding losses, and simply being successful. *PloS One* 2011; **6**: e25307.

69. Smoski MJ, Rittenberg A, Dichter GS. Major depressive disorder is characterized by greater reward network activation to monetary than pleasant image rewards. *Psychiatry Research* 2011; **194**: 263-70.

70. Samanez-Larkin GR, Wagner AD, Knutson B. Expected value information improves financial risk taking across the adult life span. *Social Cognitive and Affective Neuroscience* 2011; **6**: 207-17.

71. da Silva Alves F, Schmitz N, Figee M, et al. Dopaminergic modulation of the human reward system: a placebo-controlled dopamine depletion fMRI study. *Journal of Psychopharmacology* 2011; **25**: 538-49.

72. da Silva Alves F, Bakker G, Schmitz N, et al. Dopaminergic modulation of the reward system in schizophrenia: a placebo-controlled dopamine depletion fMRI study. *European Neuropsychopharmacology* 2013; **23**: 1577-86.

73. van Duin EDA, Goossens L, Hernaus D, et al. Neural correlates of reward processing in adults with 22q11 deletion syndrome. *Journal of Neurodevelopmental Disorders* 2016; **8**: 25.

74. Choi JS, Shin YC, Jung WH, et al. Altered brain activity during reward anticipation in pathological gambling and obsessive-compulsive disorder. *PloS One* 2012; **7**: e45938.

75. Delmonte S, Balsters JH, McGrath J, et al. Social and monetary reward processing in autism spectrum disorders. *Molecular Autism* 2012; **3**: 7.

76. Dichter GS, Kozink RV, McClernon FJ, Smoski MJ. Remitted major depression is characterized by reward network hyperactivation during reward anticipation and hypoactivation during reward outcomes. *Journal of Affective Disorders* 2012; **136**: 1126-34.

77. Dowd EC, Barch DM. Pavlovian reward prediction and receipt in schizophrenia: relationship to anhedonia. *PloS One* 2012; **7**: e35622.

78. Enzi B, Edel MA, Lissek S, et al. Altered ventral striatal activation during reward and punishment processing in premanifest Huntington's disease: a functional magnetic resonance study. *Experimental Neurology* 2012; **235**: 256-64.

79. Esslinger C, Englisch S, Inta D, et al. Ventral striatal activation during attribution of stimulus saliency and reward anticipation is correlated in unmedicated first episode schizophrenia patients. *Schizophrenia Research* 2012; **140**: 114-21.

80. Juckel G, Friedel E, Koslowski M, et al. Ventral striatal activation during reward processing in subjects with ultra-high risk for schizophrenia. *Neuropsychobiology* 2012; **66**: 50-6.

81. Morris RW, Vercammen A, Lenroot R, et al. Disambiguating ventral striatum fMRI-related BOLD signal during reward prediction in schizophrenia. *Molecular Psychiatry* 2012; **17**: 235, 80-9.

82. Nielsen MO, Rostrup E, Wulff S, et al. Alterations of the brain reward system in antipsychotic naive schizophrenia patients. *Biological Psychiatry* 2012; **71**: 898-905.

83. Plichta MM, Schwarz AJ, Grimm O, et al. Test-retest reliability of evoked BOLD signals from a cognitive-emotive fMRI test battery. *NeuroImage* 2012; **60**: 1746-58.

84. Puschmann S, Brechmann A, Thiel CM. Learning-dependent plasticity in human auditory cortex during appetitive operant conditioning. *Human Brain Mapping* 2013; **34**: 2841-51.

85. Stoy M, Schlagenhauf F, Sterzer P, et al. Hyporeactivity of ventral striatum towards incentive stimuli in unmedicated depressed patients normalizes after treatment with escitalopram. *Journal of Psychopharmacology* 2012; **26**: 677-88.

86. Weis T, Puschmann S, Brechmann A, Thiel CM. Effects of L-dopa during auditory instrumental learning in humans. *PloS One* 2012; **7**: e52504.

87. Balodis IM, Kober H, Worhunsky PD, et al. Monetary reward processing in obese individuals with and without binge eating disorder. *Biological Psychiatry* 2013; **73**: 877-86.

88. Edel MA, Enzi B, Witthaus H, et al. Differential reward processing in subtypes of adult attention deficit hyperactivity disorder. *Journal of Psychiatric Research* 2013; **47**: 350-6.

89. Enzi B, Doering S, Faber C, Hinrichs J, Bahmer J, Northoff G. Reduced deactivation in reward circuitry and midline structures during emotion processing in borderline personality disorder. *World Journal of Biological Psychiatry* 2013; **14**: 45-56.

90. Kappel V, Koch A, Lorenz RC, et al. CID: a valid incentive delay paradigm for children. *Journal of Neural Transmission* 2013; **120**: 1259-70.

91. Patel KT, Stevens MC, Meda SA, et al. Robust changes in reward circuitry during reward loss in current and former cocaine users during performance of a monetary incentive delay task. *Biological Psychiatry* 2013; **74**: 529-37.

92. Plichta MM, Wolf I, Hohmann S, et al. Simultaneous EEG and fMRI reveals a causally connected subcortical-cortical network during reward anticipation. *Journal of Neuroscience* 2013; **33**: 14526-33.

93. Roiser JP, Howes OD, Chaddock CA, Joyce EM, McGuire P. Neural and behavioral correlates of aberrant salience in individuals at risk for psychosis. *Schizophrenia Bulletin* 2013; **39**: 1328-36.

94. Treadway MT, Buckholtz JW, Zald DH. Perceived stress predicts altered reward and loss feedback processing in medial prefrontal cortex. *Frontiers in Human Neuroscience* 2013; **7**: 180.

95. Balodis IM, Grilo CM, Kober H, et al. A pilot study linking reduced fronto-Striatal recruitment during reward processing to persistent bingeing following treatment for binge-eating disorder. *International Journal of Eating Disorders* 2014; **47**: 376-84.

96. Boecker R, Holz NE, Buchmann AF, et al. Impact of early life adversity on reward processing in young adults: EEG-fMRI results from a prospective study over 25 years. *PloS One* 2014; **9**: e104185.

97. Grimm O, Heinz A, Walter H, et al. Striatal response to reward anticipation: evidence for a systems-level intermediate phenotype for schizophrenia. *JAMA Psychiatry* 2014; **71**: 531-9.

98. Pfabigan DM, Seidel EM, Sladky R, et al. P300 amplitude variation is related to ventral striatum BOLD response during gain and loss anticipation: an EEG and fMRI experiment. *NeuroImage* 2014; **96**: 12-21.

99. Rademacher L, Salama A, Grunder G, Spreckelmeyer KN. Differential patterns of nucleus accumbens activation during anticipation of monetary and social reward in young and older adults. *Social Cognitive and Affective Neuroscience* 2014; **9**: 825-31.

100. Spati J, Chumbley J, Brakowski J, et al. Functional lateralization of the anterior insula during feedback processing. *Human Brain Mapping* 2014; **35**: 4428-39.

101. de Leeuw M, Kahn RS, Vink M. Fronto-striatal dysfunction during reward processing in unaffected siblings of schizophrenia patients. *Schizophrenia Bulletin* 2015; **41**: 94-103.

102. Enzi B, Lissek S, Edel MA, et al. Alterations of monetary reward and punishment processing in chronic cannabis users: an FMRI study. *PloS One* 2015; **10**: e0119150.

103. Hagele C, Schlagenhauf F, Rapp M, et al. Dimensional psychiatry: reward dysfunction and depressive mood across psychiatric disorders. *Psychopharmacology* 2015; **232**: 331-41.

104. Hanssen E, van der Velde J, Gromann PM, et al. Neural correlates of reward processing in healthy siblings of patients with schizophrenia. *Frontiers in Human Neuroscience* 2015; **9**: 504.

105. Metereau E, Dreher JC. The medial orbitofrontal cortex encodes a general unsigned value signal during anticipation of both appetitive and aversive events. *Cortex* 2015; **63**: 42-54.

106. Redlich R, Dohm K, Grotegerd D, et al. Reward Processing in Unipolar and Bipolar Depression: A Functional MRI Study. *Neuropsychopharmacology* 2015; **40**: 2623-31.

107. Spaniol J, Bowen HJ, Wegier P, Grady C. Neural responses to monetary incentives in younger and older adults. *Brain Research* 2015; **1612**: 70-82.

108. Subramaniam K, Hooker CI, Biagianti B, Fisher M, Nagarajan S, Vinogradov S. Neural signal during immediate reward anticipation in schizophrenia: Relationship to real-world motivation and function. *NeuroImage Clinical* 2015; **9**: 153-63.

109. Balodis IM, Kober H, Worhunsky PD, et al. Neurofunctional Reward Processing Changes in Cocaine Dependence During Recovery. *Neuropsychopharmacology* 2016; **41**: 2112-21.

110. Kirschner M, Hager OM, Bischof M, et al. Deficits in context-dependent adaptive coding of reward in schizophrenia. *NPJ Schizophrenia* 2016; **2**: 16020.

111. Mori A, Okamoto Y, Okada G, et al. Behavioral activation can normalize neural hypoactivation in subthreshold depression during a monetary incentive delay task. *Journal of Affective Disorders* 2016; **189**: 254-62.

112. Vink M, de Leeuw M, Pouwels R, van den Munkhof HE, Kahn RS, Hillegers M. Diminishing striatal activation across adolescent development during reward anticipation in offspring of schizophrenia patients. *Schizophrenia Research* 2016; **170**: 73-9.

113. Garrison KA, Yip SW, Balodis IM, Carroll KM, Potenza MN, Krishnan-Sarin S. Reward-related frontostriatal activity and smoking behavior among adolescents in treatment for smoking cessation. *Drug and Alcohol Dependence* 2017; **177**: 268-76.

114. Richey JA, Ghane M, Valdespino A, et al. Spatiotemporal dissociation of brain activity underlying threat and reward in social anxiety disorder. *Social Cognitive and Affective Neuroscience* 2017; **12**: 81-94.

115. Verdejo-Roman J, Fornito A, Soriano-Mas C, Vilar-Lopez R, Verdejo-Garcia A. Independent functional connectivity networks underpin food and monetary reward sensitivity in excess weight. *NeuroImage* 2017; **146**: 293-300.

116. Kocsel N, Szabo E, Galambos A, et al. Trait Rumination Influences Neural Correlates of the Anticipation but Not the Consumption Phase of Reward Processing. *Frontiers in Behavioral Neuroscience* 2017; **11**: 85.

117. Kocsel N, Galambos A, Szabo E, et al. Altered neural activity to monetary reward/loss processing in episodic migraine. *Scientific Reports* 2019; **9**: 5420.

118. Dubol M, Trichard C, Leroy C, et al. Dopamine Transporter and Reward Anticipation in a Dimensional Perspective: A Multimodal Brain Imaging Study. *Neuropsychopharmacology* 2018; **43**: 820-7.

119. Held-Poschardt D, Sterzer P, Schlagenhauf F, et al. Reward and loss anticipation in panic disorder: An fMRI study. *Psychiatry Research: Neuroimaging* 2018; **271**: 111-7.

120. Li Z, Yan C, Lv QY, et al. Striatal dysfunction in patients with schizophrenia and their unaffected first-degree relatives. *Schizophrenia Research* 2018; **195**: 215-21.

121. Spetter MS, Feld GB, Thienel M, Preissl H, Hege MA, Hallschmid M. Oxytocin curbs calorie intake via food-specific increases in the activity of brain areas that process reward and establish cognitive control. *Scientific reports* 2018; **8**: 2736.

122. Yousuf M, Heldmann M, Gottlich M, Munte TF, Donamayor N. Neural processing of food and monetary rewards is modulated by metabolic state. *Brain Imaging and Behavior* 2018; **12**: 1379-92.

123. Just AL, Meng C, Smith DG, Bullmore ET, Robbins TW, Ersche KD. Effects of familial risk and stimulant drug use on the anticipation of monetary reward: an fMRI study. *Translational Psychiatry* 2019; **9**: 65.

124. Mori A, Klobl M, Okada G, et al. Predicting Ventral Striatal Activation During Reward Anticipation From Functional Connectivity at Rest. *Frontiers in Human Neuroscience* 2019; **13**: 289.

**2. Funnel plots for clusters in the anticipation meta-analysis**
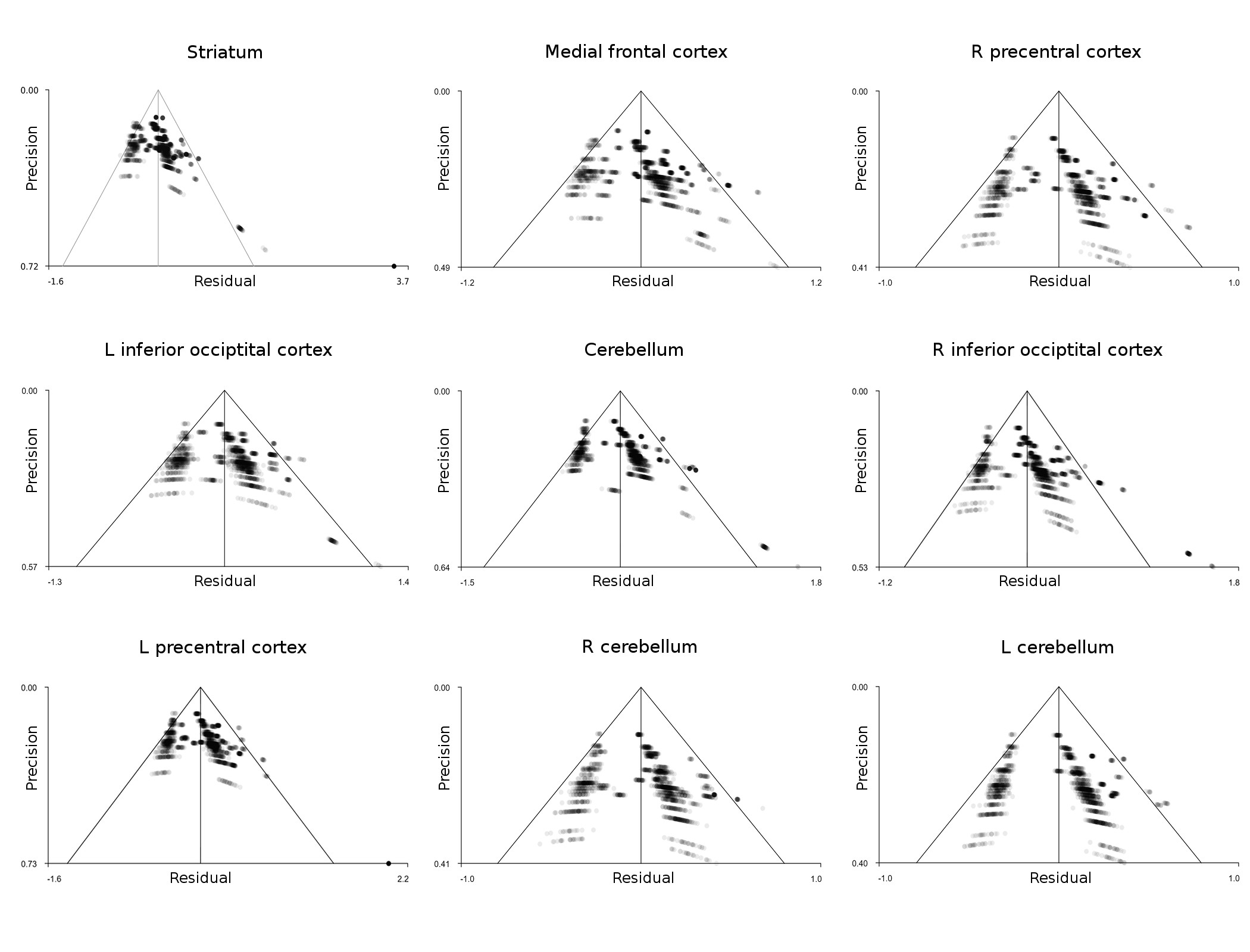


*Note:* Studies in which the effect sizes were known (e.g. there was a peak in that voxel) or in which imputations were very similar appear as a solid black point, whereas studies in which the imputations were less similar appear as a line of blurred gray points. These lines of gray points might be interpreted as the set of more likely residuals for the corresponding study.

**3. Funnel plots for clusters in the delivery meta-analysis**


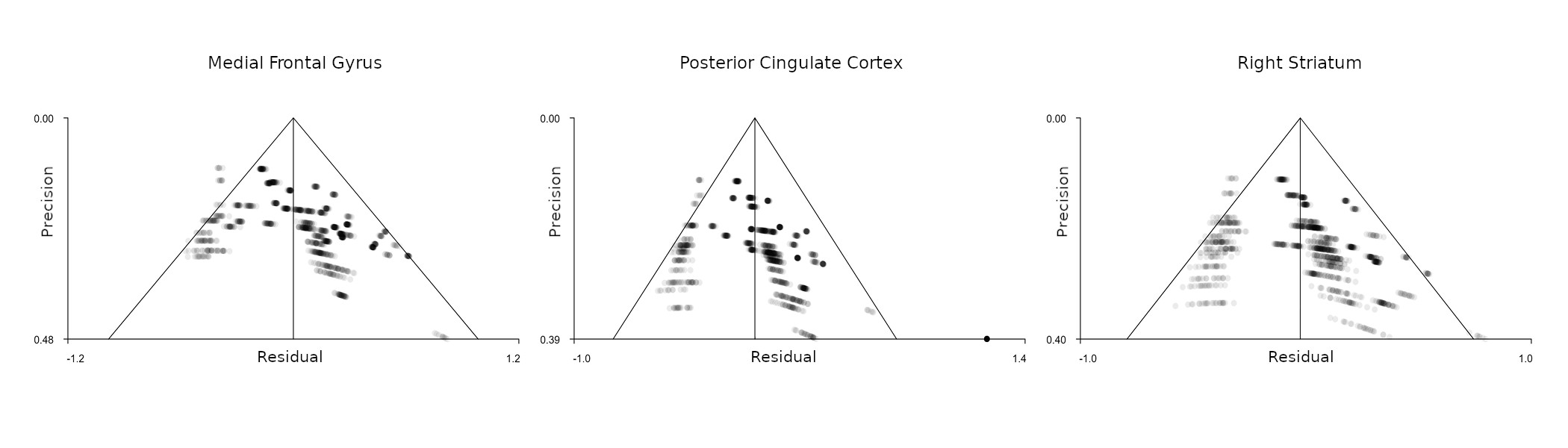


See above figure for interpretation of black and gray points.

**3. Meta-analysis results at a lower threshold of (FWER < 0.05) and extension threshold of 10 voxels.**

|  | MNI | Hedges’ *g* | Z-value | FWER |  | Voxels |
| --- | --- | --- | --- | --- | --- | --- |
|  | | | | | | |
| *Reward anticipation* | | | | | | |
|  | |  |  |  |  |  |
| Striatum | 16,6,-12 | 0.42 | 8.9 | <0.001 |  | 5644 |
|  |  |  |  |  |  |  |
| Medial frontal cortex | 0,6,34 | 0.4 | 10.4 | <0.001 |  | 3961 |
|  |  |  |  |  |  |  |
| Right pecentral cortex | 44,-20,48 | 0.29 | 7 | <0.001 |  | 667 |
|  |  |  |  |  |  |  |
| Left inferior occipital cortex | -20,-96,-16 | 0.28 | 6.2 | <0.001 |  | 633 |
|  |  |  |  |  |  |  |
| Cerebellum | 8,-62,-14 | 0.3 | 6.6 | <0.001 |  | 623 |
|  |  |  |  |  |  |  |
| Right inferior occipital cortex | 38,-86,-8 | 0.33 | 7.1 | <0.001 |  | 315 |
|  |  |  |  |  |  |  |
| Left precentral cortex | -34,-6,56 | 0.28 | 6.3 | <0.001 |  | 289 |
|  |  |  |  |  |  |  |
| Right cerebellum | 24,-46,-30 | 0.26 | 6.4 | <0.001 |  | 192 |
|  |  |  |  |  |  |  |
| Left cerebellum | -28,-68,-32  -20,-62,-24 | 0.25  0.22 | 5.6  5.6 | <0.001  <0.005 |  | 157 |
|  |  |  |  |  |  |  |
| Left superior temporal cortex | -56,-32,20 | 0.25 | 6.1 | <0.001 |  | 57 |
|  |  |  |  |  |  |  |
| Right superior occipital cortex | 14,-90,12 | 0.26 | 5.5 | <0.001 |  | 35 |
|  |  |  |  |  |  |  |
| Left middle frontal cortex | -28,46,26 | 0.24 | 5.7 | <0.001 |  | 33 |
|  |  |  |  |  |  |  |
| Left interior parietal cortex | -44,-32,42 | 0.23 | 5.1 | <0.001 |  | 21 |
|  |  |  |  |  |  |  |
| Right middle frontal cortex | 32,52,24 | 0.22 | 5.2 | <0.001 |  | 13 |
|  |  |  |  |  |  |  |
| Left calcarine fissure | 4,-90,12 | 0.21 | 5 | <0.005 |  | 12 |
|  |  |  |  |  |  |  |
| Left insula | -32,14,-14 | 0.29 | 5 | <0.005 |  | 10 |
|  |  |  |  |  |  |  |
|  | | | | | | |
| *Reward delivery* | | | | | | |
|  |  |  |  |  |  |  |
| Medial frontal cortex | 0,36,-16 | 0.44 | 8.8 | <0.001 |  | 2822 |
|  |  |  |  |  |  |  |
| Posterior cingulate cortex | 0,-26,34 | 0.37 | 7 | <0.001 |  | 1128 |
|  |  |  |  |  |  |  |
| Striatum | 10,14,-6 | 0.35 | 6.9 | <0.001 |  | 219 |
|  |  |  |  |  |  |  |
| Right angular cortex | 40,-68,46 | 0.26 | 5.1 | <0.001 |  | 14 |
|  |  |  |  |  |  |  |
